# DNA sensing in cancer: Pro-tumour and anti-tumour functions of cGAS–STING signalling

**DOI:** 10.1042/EBC20220241

**Published:** 2023-09-28

**Authors:** Otto P.G. Wheeler, Leonie Unterholzner

**Affiliations:** Division of Biomedical and Life Sciences, Faculty of Health and Medicine, Lancaster University, Lancaster, U.K.

**Keywords:** cancer, cGAS, DNA sensing, immunology, innate immunity, STING

## Abstract

The DNA sensor cGAS (cyclic GMP-AMP synthase) and its adaptor protein STING (Stimulator of Interferon Genes) detect the presence of cytosolic DNA as a sign of infection or damage. In cancer cells, this pathway can be activated through persistent DNA damage and chromosomal instability, which results in the formation of micronuclei and the exposure of DNA fragments to the cytosol. DNA damage from radio- or chemotherapy can further activate DNA sensing responses, which may occur in the cancer cells themselves or in stromal and immune cells in the tumour microenvironment (TME). cGAS–STING signalling results in the production of type I interferons, which have been linked to immune cell infiltration in ‘hot’ tumours that are susceptible to immunosurveillance and immunotherapy approaches. However, recent research has highlighted the complex nature of STING signalling, with tumours having developed mechanisms to evade and hijack this signalling pathway for their own benefit. In this mini-review we will explore how cGAS–STING signalling in different cells in the TME can promote both anti-tumour and pro-tumour responses. This includes the role of type I interferons and the second messenger cGAMP in the TME, and the influence of STING signalling on local immune cell populations. We examine how alternative signalling cascades downstream of STING can promote chronic interferon signalling, the activation of the transcription factor nuclear factor kappa-light-chain-enhancer of activated B cells (NF-κB) and the production of inflammatory cytokines, which can have pro-tumour functions. An in-depth understanding of DNA sensing in different cell contexts will be required to harness the anti-tumour functions of STING signalling.

## Introduction: DNA sensing in cancer

Our immune system has the capacity to detect and eliminate cancer cells, despite the fact that they are derived from our own body. This is partly because cancerous and pre-cancerous cells – particularly those with a high mutational burden – express proteins that can be detected as neo-antigens by the cells of our adaptive immune system [1,2]. However, a pre-requisite for effective immune activation is a favourable tumour microenvironment (TME), which relies on the innate immune mechanisms that result in the secretion of interferons, cytokines and chemokines to promote the infiltration and activation of immune cells with anti-cancer properties. One innate immune signalling axis, which can shape the immunological properties of the TME is the signalling pathway involving the adaptor protein STING (Stimulator of Interferon Genes) which is activated by the DNA sensor cGAS (cyclic GMP-AMP synthase) after the detection of cytosolic DNA or DNA damage [3]. Immune cells within the TME, stromal cells and cancer cells themselves can contribute to the DNA sensing response in cancer. Emerging evidence highlights a multi-faceted role of STING signalling in cancer, which can lead to both anti-tumour and pro-tumour signalling outcomes. This review will provide an overview of the recent advances in our understanding of how STING signalling influences anti-tumour immune responses, how this signalling can be evaded by tumours and how it can be subverted in some cancers to aid tumour progression.

### Sources of cytosolic DNA in cancer

**Figure 1:**
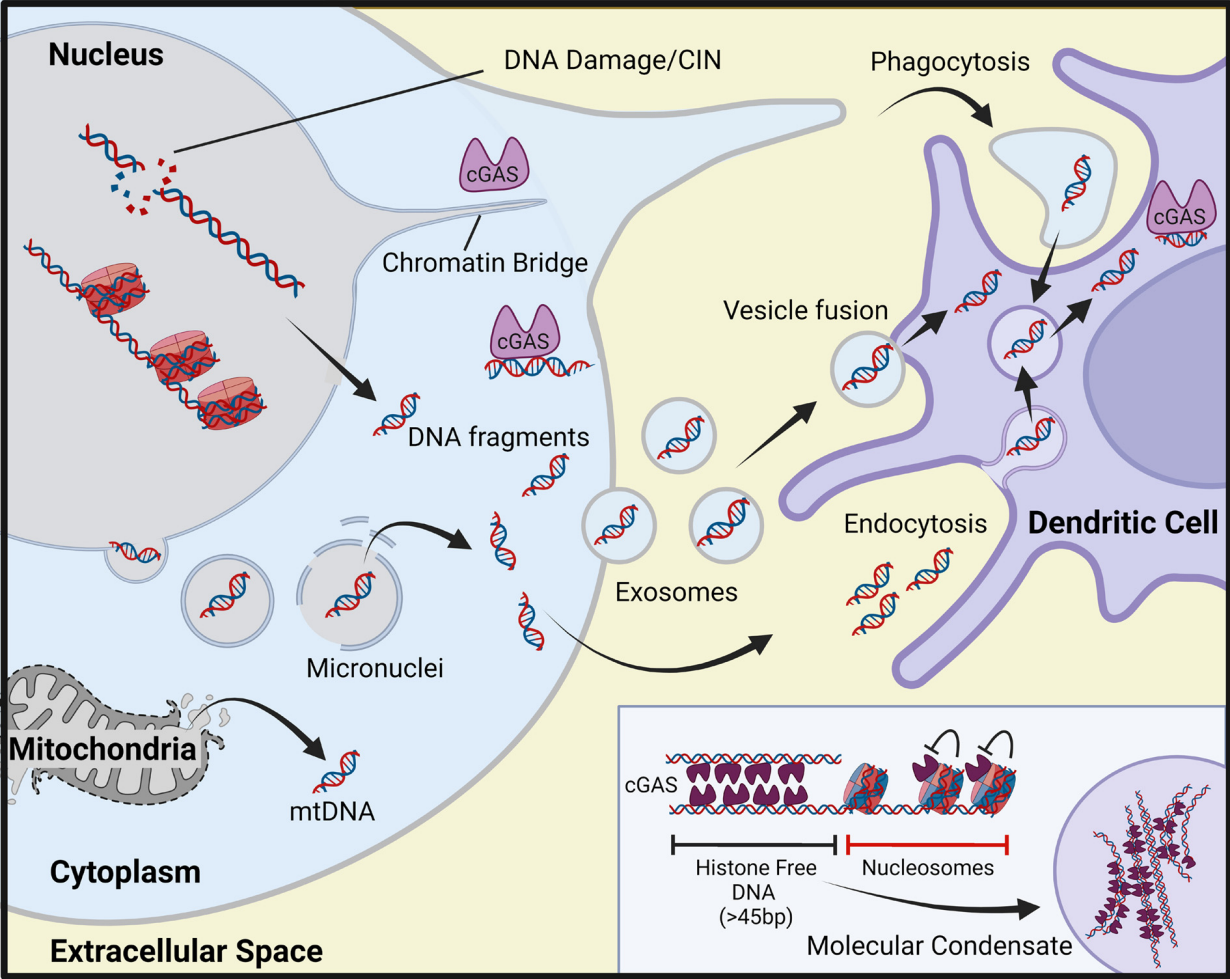
Sources of immunostimulatory DNA in tumour cells and the tumour microenvironment The presence of self-DNA within the cytosol can occur due to chromosomal instability in cancer cells as well as from additional DNA damage caused by cancer therapies. Micronuclei are a source of self-DNA within the cytosol often naturally arising in metastatic and genomically unstable cancer cells as well as from exposure to ionising radiation. Chromatin bridges contain DNA connecting two daughter cells. Mitochondrial (mt) DNA is another potential source of immunostimulatory DNA in the cytosol which arises from mitochondrial damage. cGAS can only bind stretches of unmodified double-stranded DNA greater than 45 bp, its activation is inhibited by nucleosomes. cGAS molecules assemble on histone free DNA forming a ladder-like structure and can form higher order structures which agglomerate into molecular condensates which enhance the production of cGAMP. Nearby immune cells can take up cGAMP or DNA released into the TME by dead or dying tumour cells. Tumour cells can also export DNA into the TME in membrane bound vesicles such as exosomes which are taken up by local immune cells to activate STING signalling.

Cytosolic DNA is detected as pathogen-associated molecular pattern (PAMP), during viral infection for instance, but it can also serve as danger- or damage-associated molecular pattern (DAMP) when the cell’s own nuclear or mitochondrial DNA is damaged and gains access to the cytosol. Tumour cells often contain cytosolic DNA, due to persistent DNA damage and chromosomal instability (CIN), which is a hallmark of cancer [4,5], and additional DNA damage is induced by radiotherapy and chemotherapy with DNA damaging agents. The presence of CIN and DNA breaks is associated with the formation of micronuclei – membrane bound structures that form around damaged DNA and lagging/mis-segregated chromosomes during mitotic exit [6]. Upon rupture of their unstable membrane micronuclei expose their DNA content to the cytosol [7–11]. DNA damage and CIN may also lead to the direct leakage of DNA fragments out of the nucleus and the formation of chromatin bridges which can also be detected by cytosolic DNA sensors [12,13] (Figure 1). Furthermore, leakage of DNA from damaged mitochondria can also contribute to the innate immune activation [14].

### DNA sensing by cGAS and STING

**Figure 2:**
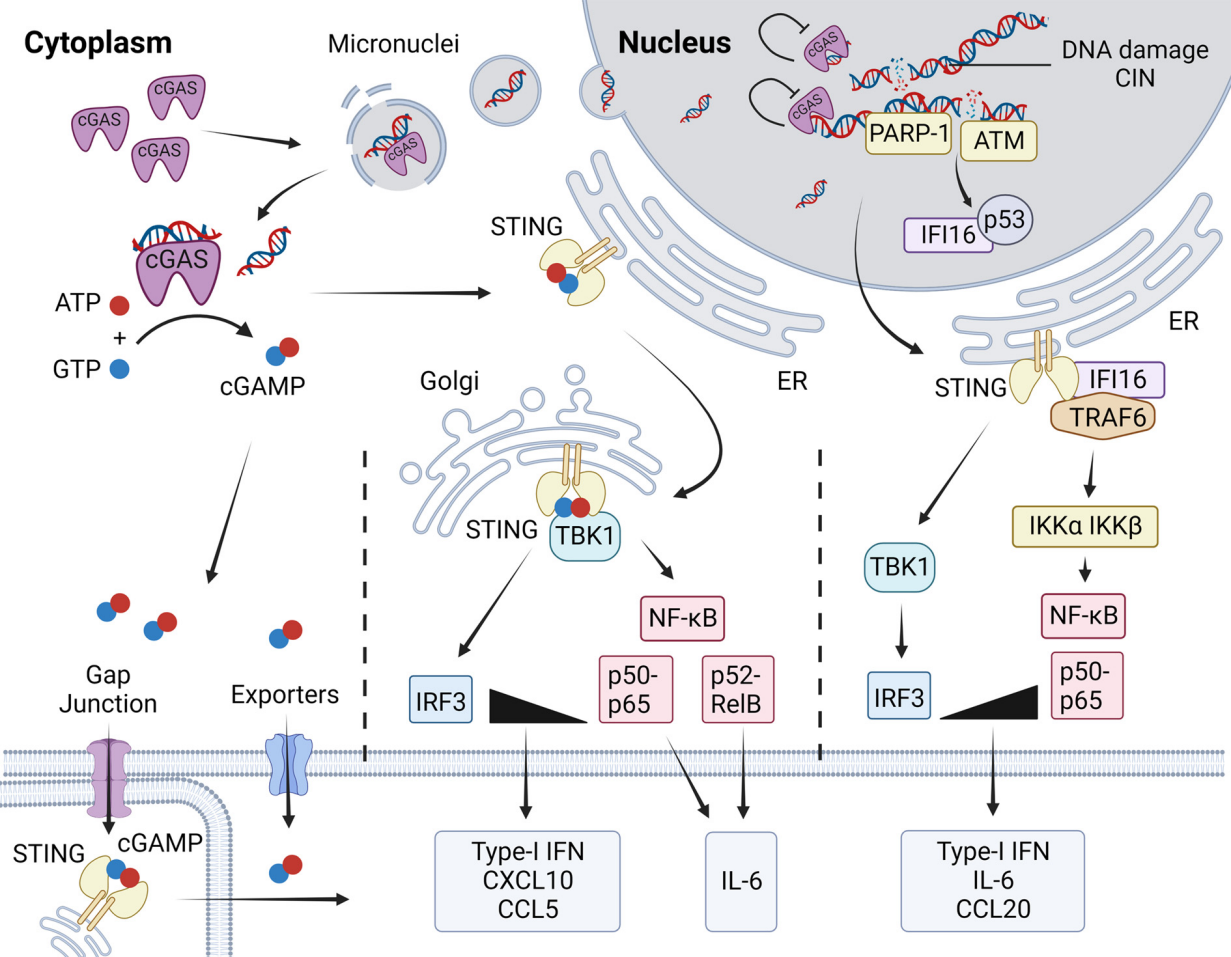
Innate immune signalling in response to cytosolic DNA and DNA damage On detecting cytosolic DNA, cGAS synthesises the second messenger cGAMP which can be exported to adjacent cells or the extracellular environment using gap junctions and exporters. Within the cell, cGAMP can go on to bind STING anchored to the endoplasmic reticulum (ER). STING is subject to numerous posttranslational modifications, then transported from the ER to the Golgi where it forms a complex with TBK1 and is phosphorylated by TBK1 at Serine 366. This serves to recruit the transcription factor IRF3 to the complex, which is also phosphorylated by TBK1. IRF3 and NF-κB p65 then promote the production of IFN-Is and chemokines including CXCL10 and CCL5. An alternative non-canonical STING signalling pathway has been identified in human epithelial cells. This pathway does not require the activity of cGAS, but the DNA damage sensing proteins PARP-1 and ATM, as well as the DNA binding protein IFI16 which shuttles between the nucleus and cytosol. cGAS-independent DNA damage sensing favours the activation of NF-κB over IRF3, generating a more pro-inflammatory cytokine profile that is distinct from canonical STING signalling. In cancer cells, canonical STING activation can be re-wired to induce the activation of NF-κB p52, which also induces a pro-inflammatory cytokine profile that may aid tumour progression.

The DNA sensor cGAS and its adaptor protein STING constitute the key signalling axis for sensing dsDNA within the cytosol of many different cell types (Figure 2) [15–19]. cGAS binds double-stranded (ds) DNA in a sequence-independent manner, and cGAS dimers form ladder-like assemblies with dsDNA in higher order signalling domains in the cytosol [20–24]. While cGAS is also present in the nucleus of many cells, its catalytic activity is inhibited by interaction with nucleosomes on chromatin (Figure 1) [25–29]. Independent of its catalytic activity cGAS has also been shown to interact with DNA replication forks, regulate genomic stability as well as inhibit homologous recombination [30–32]. Upon detection of dsDNA in the cytosol, cGAS catalyses the synthesis of the second messenger 2′3′-cyclic GMP-AMP (cGAMP), which then binds STING, a membrane protein residing at the endoplasmic reticulum (ER) [16–18,33–35]. Activated STING dimers translocate through the ER–Golgi intermediate compartments (ERGIC) to the Golgi apparatus [36–39]. STING forms a complex with TANK-binding kinase 1 (TBK1) which phosphorylates STING and the transcription factor interferon regulatory factor (IRF3) as well as activating nuclear factor kappa-light-chain-enhancer of activated B cells (NF-κB) (Figure 2) [15,39–41]. These transcription factors go on to promote the expression of type I interferons (IFN-I), cytokines and chemokines including CXCL10 and CCL5. STING activation can also induce autophagy, senescence and cell death, depending on signal strength and cell context [42–44]. STING-mediated activation of inflammasome complexes has also been described [45], but other DNA sensors such as AIM2 (Absent in Melanoma 2) may also play an important role in inflammasome activation, depending on cell context [46,47].

cGAS-STING signalling is tightly regulated by post-translational modifications, including ubiquitylation with K48-, K63-, K27- and K11-linked ubiquitin chains, modification with ubiquitin-like proteins as well modification with metabolites and lipids [45,47]. Additional DNA binding proteins have also been shown to synergise with cGAS in the activation of STING. This includes Interferon-γ-Inducible Protein 16 (IFI16), the helicase DDX41, the DNA damage response kinase DNA-PK and the Z DNA-binding protein ZBP1 [51–57]. It remains to be elucidated whether additional DNA binding proteins contribute specificity to the sensing of DNA ligands, or whether they represent additional fail-safe mechanisms for the full activation of DNA sensing responses only when appropriate. Antagonistic and synergistic cross-talk between DNA sensing pathways such as cGAS-STING signalling and inflammasome activation has been reported [58–60], which further influences downstream signalling outcomes.

STING can also be activated independently of cGAS, such as following membrane fusion or after uptake of cGAMP from neighbouring cells [61,58]. Bacterial cyclic di-nucleotides (CDNs) can also activate STING directly in response to infection [59]. While cGAMP sensing by STING bypasses the DNA sensing role of cGAS in bystander cells, a non-canonical involvement of cGAS in the detection of extracellular cGAMP has also been described [62]. cGAS-independent STING activation following the detection of nuclear DNA damage has also been described in human epithelial cells, which may be of particular relevance to cancer. This alternative STING activation pathway involves the DNA binding protein IFI16, as well as the DNA damage sensing factors ATM, PARP1 and p53 [60]. The cGAS-independent activation of STING links the nuclear DNA damage response to innate immune activation prior to the formation of micronuclei and results in the expression of a different – more pro-inflammatory – set of cytokines and chemokines [60].

## Anti-tumour functions of STING signalling

**Figure 3:**
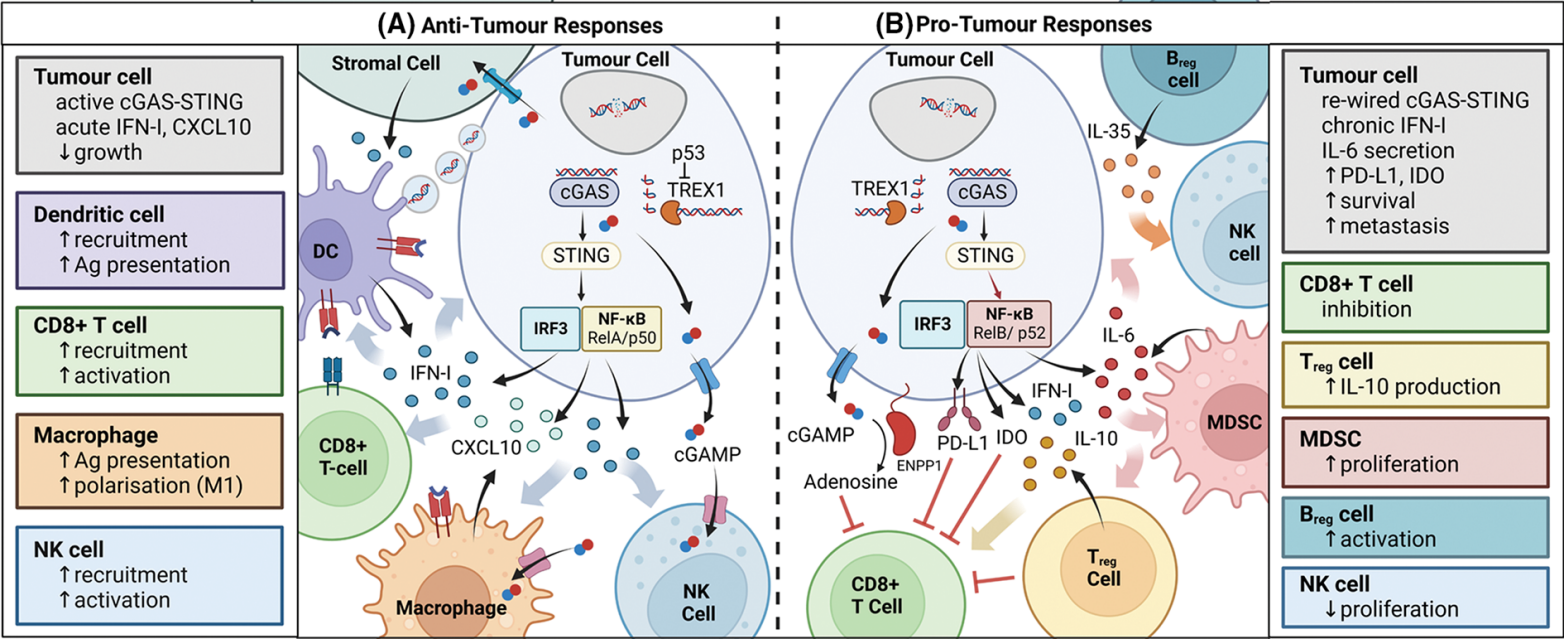
Pro-tumour and anti-tumour responses of STING signalling in cancer (**A**) Anti-tumour responses. The cGAS-STING signalling axis with IFN-I and CXCL10 secretion as signalling output co-ordinates multiple anti-tumour functions. The tumour suppressor p53 negatively regulates TREX1, making cytosolic DNA available for detection by cGAS. Tumour DNA released into the TME can be taken up by DCs promoting their activation. cGAMP can also be transported via gap junctions directly to adjacent cells or into the TME where it is taken up by local immune cells including NK cells and macrophages. The uptake of cGAMP by the importers of these cells can trigger their own STING signalling cascades and the production of IFN-Is. IFN-I responses enhance the activity of cytotoxic T-cells, DCs and NK cells as well as promote M2 to M1 macrophage polarisation. The activation of DCs is particularly important for generating adaptive CD8+ T-cell responses. (**B**) **Pro-tumour responses.** Chronic IFN-β signalling can promote tumour growth and immunosuppression. This signalling can trigger the expression of a subset of ISGs known as the IFN-related DNA damage resistance signature (IRDS) with potentially pro-tumour functions. IFN-I and IFN-II signalling has been linked to the up-regulation of PD-L1/2 and IDO which have immunosuppressive properties. STING signalling can also be subverted in some cancers to promote the activation of the non-canonical family of NF-κB transcription factors (p52/RelB) which result in the expression of IL-6. IL-6 is important for cancer cell survival and metastasis, and a pro-inflammatory TME can suppress anti-tumour immune responses through the recruitment of MDSC and regulatory T cells. cGAMP can be exported into the TME where it can be broken down and converted to immunosuppressive adenosine by ENPP1. cGAMP can trigger apoptosis and impair proliferation in T-cells which express high levels of STING. STING signalling in regulatory B cells can also impair NK cell function via IL-35 secretion.

cGAS–STING signalling has been shown to have potent anti-cancer activities in a variety of mouse tumour models (Figure 3A). The production of STING-induced IFN-Is and chemokines by immune cells in the TME and by the cancer cells themselves facilitates further immune cell infiltration and the promotion of adaptive anti-cancer immune responses [9,11,63–66]. Furthermore the induction of cGAS–STING signalling in cancer treatments such as radiotherapy, is also thought to enable the generation of spontaneous adaptive anti-tumour immunity [66–70]. This has led to a significant interest in harnessing cGAS–STING signalling in cancer therapy and the development of STING agonists [63,65,66,71–76].

### STING signalling in cancer cells

The first potential point of activation of cGAS–STING pathway is within the cancer cells themselves following detection of cytosolic DNA from micronuclei for instance [7,8]. This may be a result of inherent CIN or further DNA damage from cancer therapy. Cancer cells can contribute to the anti-tumour responses generated against them through the secretion of IFN-Is and chemokines into the TME [9,11,63,77–79]. In that way, the STING signalling capacity of tumour cells themselves may shape their TME, with IFN-I signalling being associated with ‘hot’ tumours containing infiltrating DCs and T cells. Moreover, following cell-intrinsic DNA sensing, cancer cells can also export the second messenger cGAMP directly into neighbouring immune and stromal cells in the TME. Gap junctions between cells have been shown to facilitate the transfer of cGAMP between tumour and stromal cells to promote IFN-I responses in neighbouring cells [58,80,81]. cGAMP can also be secreted into the extracellular space. Several transporters, channels and pumps which shuttle cGAMP between the extracellular environment and the cytosol have recently been identified and may contribute to the propagation of STING signalling in the TME [62,82–87].

### STING signalling in endothelial cells

cGAMP production and export by tumour cells can activated STING signalling in neighbouring endothelial cells, to induce further IFN-β and CXCL10 production in the TME. Activation of STING within the endothelium was also shown to be accompanied by increased expression of factors associated with T-cell adhesion to the endothelium and the infiltration of lymphocytes into the TME. The activation of STING in endothelial cells in the tumour vasculature is also thought to protect against angiogenesis and promote vascular normalisation in tumours [63–65,88].

### STING signalling in immune cells

Immune cells in the TME can respond to extracellular tumour-derived DNA and activate the DNA sensing pathway. The uptake of tumour DNA has been shown to be particularly important for activating DCs in the TME [68,69,89]. How precisely the uptake of DNA from the TME in these instances occurs is uncertain. Extracellular tumour DNA may be available following cancer cell death, and uptake may be facilitated by association with chromatin modifying proteins such as HMGB1, or through fusion of DNA-containing extracellular vesicles [68,69,89–95]. Following the uptake of extracellular DNA in the cytosol of myeloid cells, cGAS is activated leading to IFN-I production. In mice CD11c+ DCs were found to be one of the main producers of STING-dependent IFN-Is in the TME [68–70]. STING-induced type I interferons (IFN-Is) can further stimulate tumour infiltrating dendritic cells (DCs) and promote the presentation of tumour antigens to CD8+ T cells [96,97]. Anti-tumour adaptive immune responses are lost in mouse hosts lacking the IFN-I receptor or STING in DCs, rendering them unable to reject implanted tumours [70,97,97]. Conversely the intra-tumoral injection of STING agonists leads to immune-mediated clearance of tumours in mice, showing that activation of STING signalling in both tumour cells and immune cells may contribute to tumour clearance [81,98,99]. STING signalling has also shown to play an important role in regulating the polarisation of macrophages to an M1 anti-tumour phenotype in some tumours [99,100]. The human importer SLC46A2 facilitates the uptake of cGAMP by human macrophages and monocytes where it activates STING signalling [84]. Similarly, STING-dependent IFN-β production has been shown to be important for promoting NK activity in mouse models of glioblastoma, lymphoma and melanoma tumour [101,102]. STING signalling is also thought to reduce numbers of myeloid-derived suppressor cells in the TME [103,104], although this may not be the case in all tumours [105,106]. The STING-induced production of chemokines such as CXCL9, CXCL10 and CCL5 promote further recruitment of immune cells into the TME, and high levels of STING expression in tumours has been linked to overall higher immune cell infiltration in cancers [107].

## STING agonists as anti-tumour agents

Activation of STING signalling has been shown to be a feature of ‘hot’ tumours, which show infiltration of DCs and cytotoxic T cells – a pre-requisite for detection of tumour antigens by adaptive immune responses and for effective immunotherapy [108]. Thus, there has been considerable interest in utilising STING agonists as potential anti-cancer therapies. Numerous studies have investigated using STING agonists such as cGAMP, other cyclic di-nucleotides or small molecule activators of STING in mouse tumour models [71–76,99,109]. There is a large body of evidence that STING agonists have dramatic anti-tumour effects in these systems, enabling tumour clearance and often protecting against subsequent challenge with tumour cells. However, so far none of these successes have translated into an effective therapy in clinical trials [110]. While the lack of success with an early STING agonist, DMXAA, was later shown to be due to its specificity for murine STING [111,112], it is unclear what limits the efficacy of other STING agonists in patients. It remains to be investigated whether this is due to limitations in agonist activity and bio-availability (which could be overcome through advances in drug design and delivery) or due to more fundamental mechanistic limitations which would be more difficult to address. Underlying reasons may be mouse–human species differences in STING function or the dynamic re-wiring of STING signalling in human tumours.

## Evasion of STING signalling during tumour progression

Due to of the anti-tumour effects of STING signalling, it was originally proposed that the pathway must be dysfunctional or epigenetically suppressed in cancer cells [113]. Although this has been demonstrated in several cancer cell lines, the genes for either cGAS or STING are rarely mutated in cancers [4,113–115]. Similarly, while STING and cGAS expression have been found to be epigenetically silenced in some tumours, many cancers show normal or elevated cGAS and STING expression [114–118]. The sensing of cytosolic DNA can also be attenuated through the over-expression of the cytosolic DNase TREX1 or extracellular DNases, and the inhibition of DNA uptake into DCs [79,94,118–120]. Analogously, cGAMP in the extracellular environment can be broken down by ectonucleotide pyrophosphate phosphodiesterase 1 (ENPP1) found on cell surfaces [83,121]. It has been shown that ENPP1 is increasingly up-regulated as cancer cells become metastatic, and this enhances cancer cell migration in a cGAS-dependent manner [122]. In addition to preventing immune cell activation by removal of extracellular cGAMP, the breakdown of cGAMP increases levels of adenosine which further inhibits anti-tumour immune responses [122,123]. This has generated interest in ENPP1 as therapeutic target in its own right [124].

## Pro-tumour functions of STING signalling

In addition to its potent anti-tumour activities, STING signalling has also been shown to contribute to cancer cell survival and tumour progression in some contexts. The balance of downstream STING signalling outputs may be differentially regulated in tumour cells, so that anti-tumour IFN-I and cell death responses are limited, while pro-inflammatory and pro-survival programs are promoted.

While short-lived interferon responses due to acute STING stimulation have clear anti-tumour effects mediated by IFN-Is, it has been shown that low levels of chronic IFN-β signalling can promote cancer cell survival (Figure 3B) [125–128]. Mechanistically, prolonged low levels of IFN-β signalling cause the activation of an unphosphorylated ISGF3 (U-ISGF3) complex, which promotes the expression of a subset of interferon-stimulated genes with pro-tumour functions [127–129]. Chronic IFN-I signalling and low-grade inflammation can have additional effects on the immune composition of the TME, with the recruitment of myeloid-derived suppressor cells contributing to radioresistance and tumour progression [105]. In addition to this, both type-I and -II IFNs up-regulate the expression of programmed death ligand (PD-L1/2) in cancer cells [78,130]. PD-L1 can interfere with STING-mediated IFN production and IFN-mediated cytotoxicity in cancer cells alongside its ability to repress T cell responses [128,131]. The expression of the enzyme indoleamine-2,3-dioxygenase (IDO), which promotes an immune-suppressive TME has also been linked to STING signalling [106,132,133].

### STING-dependent NF-κB activation in tumour cells

The STING-dependent expression of pro-inflammatory cytokines, and particularly IL-6, can also promote inflammation-driven tumour progression. STING-deficient mice have been found to be resistant to the development of skin tumours induced by the mutagen DMBA, which drives tumorigenesis through IL-6-mediated inflammation [134]. Analysis of transcriptomics data from the Cancer Cell Line Encyclopedia (CCLE) and The Cancer Genome Atlas (TCGA) of primary tumour samples (including pancreatic adenocarcinoma, cutaneous melanoma, prostate adenocarcinoma and breast adenocarcinoma) has also shown that higher levels of STING expression is associated with higher levels of pro-inflammatory gene expression [10]. IL-6 in particular is a key enhancer of survival and metastasis in cancer [135–138]. In triple negative breast cancer cells with CIN, the depletion of cGAS–STING resulted in a reduction in IL-6 production and impaired cancer cell survival [139].

The expression of pro-inflammatory cytokines such as IL-6 is driven by the NF-kB family of transcription factors. In most cells, canonical cGAS–STING signalling induces only modest levels of NF-κB p65 activation, through signalling by TBK1 which favours the activation of the transcription factor IRF3 [41]. However, an alternative cGAS-independent mode of STING activation by nuclear DNA damage results in preferential NF-κB p65 activation and a more pro-inflammatory cytokine profile than conventional DNA sensing [60]. Thus, it is possible that this pathway contributes the production of pro-inflammatory cytokines during chronic DNA damage or CIN in cancer.

As an additional immune evasion strategy, tumour cells can also re-wire cGAS-mediated STING signalling towards increased non-canonical NF-κB activation in cancer cells through the NF-κB subunits p52 and RelB [118,140]. This drives the production of pro-inflammatory cytokines, rather than IFN-Is following DNA sensing, and thus could be a reason why cancers retain cGAS–STING signalling and subvert it to a different outcome. Non-canonical NF-κB signalling through p52 can in turn interfere with conventional STING–TBK1–IFN signalling, and this has been shown to further impair the anti-tumour effects of radiotherapy [140]. Similar inhibitory effects on STING signalling have also been attributed to IL-6 production in prostate cancer [141]. Thus, the collective evidence points towards a model where STING signalling is not restricted to interferon responses with anti-tumour functions, but rather can be adapted dynamically to produce both anti- and pro-tumour signalling outcomes during tumour progression.

### Pro-tumour functions of STING signalling in immune cells

It is becoming increasingly clear that the ultimate outcome of STING signalling is also highly sensitive to cell context and differs between different cell types. For instance, while cGAMP transport to DCs can stimulate potent anti-tumour responses, its transport into astrocytes can promote brain metastasis through the production of cytokines that are beneficial for cancer cell growth and survival [80]. Activation of STING signalling in adaptive immune cells themselves can also negatively impact anti-cancer immune responses. For instance, regulatory B cells can make use of STING–IRF3 signalling to induce the production of IL-35, which impairs NK cell-mediated anti-tumour activity [142]. Activation of STING signalling can also induce apoptosis in T cells, which may negatively impact anti-tumour responses [44,133,143–151]. Adding to the complexity, the outcomes of STING signalling in T cells are also influenced by the engagement of the T-cell receptor [133,147,151]. It has been proposed that these negative impacts of STING signalling may be overcome by the use of lower doses of STING agonists or the specific targeting to tumour cells [149,151]. However, given that activation thresholds and cell type specificities may also differ between mouse models and individual cancer patients, there is an urgent need to understand how downstream STING signalling effects are regulated in different human cell contexts.

## Conclusions and outlook

The intense study of DNA sensing pathways in the last decade or so has revealed a crucial role of the cGAS–STING–interferon signalling axis in shaping immune responses during infection, autoinflammation and cancer. However, despite the fact that the anti-tumour role of STING signalling is well documented in mouse tumour models and multiple STING agonists have been tested in clinical trials, these approaches have not yet shown efficacy as anti-tumour agents in patients. Indeed, STING signalling has been shown to have both pro- and anti-tumour functions in cancer cells, stromal cells and the immune cells in the TME. While the activation of canonical, acute STING signalling that results in IFN-I induction has been studied in great molecular detail and clearly possess anti-tumour functions, we know much less about how low-grade, chronic STING signalling is induced in response to DNA damage. We will also need to understand how different kinds of DNA damage induced by radio- and chemotherapy regimens affect DNA sensing pathways, so that we can avoid potential pro-tumour effects. There are many open questions about how STING activation drives different cellular outcomes, from the predominant activation of IRF3 to canonical or non-canonical NF-κB activation, as well as inflammasome activation and cell death or senescence pathways. In the future, it may be possible to therapeutically influence the balance of STING signalling outputs, rather than STING activation *per se*, to promote the anti-tumour functions of DNA sensing in cancer, while limiting adverse effects on tumour progression.

## Summary

- The detection of cytosolic DNA and DNA damage results in the activation of the innate immune adaptor STING in cancer cells, immune cells or endothelial cells within the tumour microenvironment. All these cell types can contribute to the production of type I interferons which have anti-tumour functions.
- If the STING signalling response is evaded or diverted towards a more pro-inflammatory cytokine profile, this can instead promote the establishment of an immune-suppressive microenvironment and can aid tumour cell survival and metastasis.
- A deeper understanding of how different cell contexts and regulatory mechanisms influence signalling outputs downstream of STING will be important for the development of STING-targeting immunotherapies.

## Open Access

Open access for this article was enabled by the participation of Lancaster University in an all-inclusive *Read & Publish* agreement with Portland Press and the Biochemical Society under a transformative agreement with JISC.

## Abbreviations

CCLE: Cancer Cell Line Encyclopedia
CIN: chromosomal instability
cGAMP: cyclic GMP-AMP
cGAS: cyclic GMP-AMP synthase
DAMP: damage-associated molecular pattern
DC: dendritic cell
ENPP1: ectonucleotide pyrophosphate phosphodiesterase 1
ER: endoplasmic reticulum
ERGIC: ER–Golgi intermediate compartment
IFI16: interferon gamma inducible protein 16
IFN-I: type I interferon
IL: interleukin
IRF3: interferon regulatory factor
ISG: interferon-stimulated gene
NF-κB: nuclear factor κ-light-chain-enhancer of activated B cell
NK: natural killer
PAMP: pathogen-associated molecular pattern
PARP: poly(ADP-ribose) polymerase
PD-L1/2: programmed death ligand
STING: STimulator of Interferon Genes
TBK1: TANK-binding kinase 1
TCGA: The Cancer Genome Atlas
TME: tumour microenvironment
TREX1: three prime repair exonuclease
ZBP-1: Z DNA binding protein 1
